# Microstructures, Mechanical Properties and Electromagnetic Wave Absorption Performance of Porous SiC Ceramics by Direct Foaming Combined with Direct-Ink-Writing-Based 3D Printing

**DOI:** 10.3390/ma16072861

**Published:** 2023-04-04

**Authors:** Jianqin Wu, Lu Zhang, Wenqing Wang, Ruyue Su, Xiong Gao, Suwen Li, Gang Wang, Rujie He

**Affiliations:** 1 Institute of Advanced Structure Technology, Beijing Institute of Technology, Beijing 100081, China; 2 Anhui Key Laboratory of High-Performance Non-Ferrous Metal Materials, Anhui Polytechnic University, Wuhu 241000, China

**Keywords:** direct foaming, direct-ink writing, mechanical properties, electromagnetic (EM) wave absorption

## Abstract

Direct-ink-writing (DIW)-based 3D-printing technology combined with the direct-foaming method provides a new strategy for the fabrication of porous materials. We herein report a novel method of preparing porous SiC ceramics using the DIW process and investigate their mechanical and wave absorption properties. We investigated the effects of nozzle diameter on the macroscopic shape and microstructure of the DIW SiC green bodies. Subsequently, the influences of the sintering temperature on the mechanical properties and electromagnetic (EM) wave absorption performance of the final porous SiC-sintered ceramics were also studied. The results showed that the nozzle diameter played an important role in maintaining the structure of the SiC green part. The printed products contained large amounts of closed pores with diameters of approximately 100–200 μm. As the sintering temperature increased, the porosity of porous SiC-sintered ceramics decreased while the compressive strength increased. The maximum open porosity and compressive strength were 65.4% and 7.9 MPa, respectively. The minimum reflection loss (*R_L_*) was −48.9 dB, and the maximum effective absorption bandwidth (EAB) value was 3.7 GHz. Notably, porous SiC ceramics after sintering at 1650 °C could meet the application requirements with a compressive strength of 7.9 MPa, a minimum R_L_ of −27.1 dB, and an EAB value of 3.4 GHz. This study demonstrated the potential of direct foaming combined with DIW-based 3D printing to prepare porous SiC ceramics for high strength and excellent EM wave absorption applications.

## 1. Introduction

The increasing intensity of military competition today has been driving the development of various high-tech military equipment such as stealth fighters, which face challenges in effectively absorbing electromagnetic (EM) waves [1,2,3]. Thus, the requirement for materials with light weight, high strength, and efficient EM wave absorption performance that can be applied at complex high temperatures is particularly urgent. Silicon carbide (SiC) ceramic is a dielectric absorber due to its inherent electric dipole polarization, standing out for its unique properties in terms of EM wave absorption [4,5,6]. Owing to its high temperature resistance and corrosion resistance, SiC ceramic can be applied to harsh working environments and has excellent EM wave absorption performance [7]. The specific morphologies and structures of SiC have been reported to enhance its EM wave absorption [8]. SiC ceramics can be in the form of wires [9,10,11] or be fibrous [12], tubular [13,14], or porous [15,16,17]. Especially due to its unique microstructure, porous SiC ceramic has excellent lightweight properties and superior impedance-matching performance, which meets the requirements of EM wave absorption applications in high temperature environments [18,19,20].

Nowadays, various mainstream methods have been widely investigated with the fabrication of porous ceramics and composites: partial sintering, replica template, sacrificial template, and direct foaming [21,22,23,24]. However, the challenges to these methods, including the complex manufacturing processes and high sintering temperatures, as well as the simple methods to shape porous SiC ceramics into complex shapes, remain unexplored [25]. It is well known that additive manufacturing (AM) technology or 3D printing is considered a new manufacturing method and has been extensively studied in many materials [26,27,28]. Among various 3D-printing technologies, extrusion-based 3D-printing methods are considered to be one of the simplest and most widely used processes [29,30]. Especially, the direct-ink-writing (DIW)-based 3D-printing technique has been successfully applied as a simple, effective, and easy process to fabricate ceramics with complex geometries [31,32,33]. However, in ensuring the shape retention of the extruded filament, it has particular requirements for the rheological properties of the ink, which should have a sufficiently high solid loading, yield stress, and elastic modulus [34]. Traditional fabrication methods of porous ceramics provide the precondition for slurry-based DIW-based 3D-printing processes to build micron-scale pores or even nanoscale pore structures, and templates or foams can be applied by conventional processes to obtain micron-scale or even nanoscale pore structures. Several studies have demonstrated that DIW-based 3D printing could be combined with conventional methods, such as sacrificial templates [35] and direct foaming [36], to fabricate porous ceramics. Particularly, the combination of DIW-based 3D printing and direct foaming is suitable for the fabrication of porous ceramics with complex shapes due to the advantages of the simple post-treatment process and the ability to produce ceramics with high porosity. Muth et al. [37] transformed the alumina sol to gelation by adjusting the pH, which was due to the effects of pH on the zeta potential of the particles, causing the colloidal particles to attract each other and form a gel network. At that point, the viscosity of the ink increased, and the elastic modulus and yield stress also increased to fulfill the requirements of the ink. Benito et al. [38] prepared binary colloidal gel foams formed by alumina and carbon particles as porogens by adjusting the pH value and investigated the effects of composition on the rheology and printing performance of the foam ink. Minas et al. [39] added n-octane to the alumina suspension modified by a propionic acid surface and obtained a stable emulsion foam suitable for DIW-based 3D printing. In addition to adjusting the pH or adding emulsifiers, there is an even more convenient way of foaming ink by adding surfactants. Guo et al. [40] fabricated printable foam ink by respectively adding polyvinyl alcohol and cetyltrimethylammonium bromide—which could reduce the surface tension of the particles—to the silica and alumina sol. These reports demonstrated that the preparation of printable silicon carbide foam ink was an implementable strategy. Ma et al. [41] also prepared porous SiC-based composites using the DIW method, using geopolymers as a binder and foaming agent. However, studies on the physical properties, such as EM wave absorption properties, of porous SIC ceramics prepared by DIW-based 3D printing have not been reported.

Consequently, based on our previous method of preparing SiC-based ceramic ink [42], we combined the above-mentioned strategies of DIW-based 3D printing and direct foaming. In this paper, we first fabricated printable SiC foam inks and then systematically investigated the effects of nozzle diameter on the formability and microscopic morphology of the SiC green samples during DIW-based 3D printing. Finally, the effects of sintering temperature on the mechanical properties and EM wave absorption performance of porous SiC-sintered ceramics were also explored. This study could demonstrate the potential of direct foaming in combination with direct-ink-writing-based 3D printing for the preparation of high-intensity electromagnetic wave-absorbing porous SiC ceramics.

## 2. Materials and Methods

### 2.1. Raw Materials

SiC powders (purity > 99%, Shanghai Aladdin Biochemical Technology Co., Ltd., Shanghai, China) with an average particle size of 0.5~0.7 μm were chosen as the main material. Yttria (Y_2_O_3_, purity > 99.9%, Beijing Meridian Technology Co., Ltd., Beijing, China), alumina (Al_2_O_3_, purity > 99.9%, Beijing Meridian Technology Co., Ltd., Beijing, China), and silica (SiO_2_, purity > 99.9%, Beijing Meridian Technology Co., Ltd., Beijing, China) powders were used as the sintering additives. Polyethylene glycol (PEG6000, Xilong Chemical Co., Ltd., Shantou, China) was employed to assure a good dispersion of the SiC powders in the ink. Methyl cellulose (MC, Shanghai Macklin Biochemical Technology Co., Ltd., Shanghai, China) was employed as the binder. Polyvinyl alcohol (PVA-205; 87–89% hydrolyzed; Shanghai Aladdin Biochemical Technology Co., Ltd., Shanghai, China) was used to prevent collapse of pore structures. Dodecayltrimethylaminium bromide (DTAB, 99%, Shanghai Meryer Chemical Technology Co., Ltd., Shanghai, China) was selected as the foaming agent.

### 2.2. Fabrication of Porous SiC Ceramics

**Figure 1:**
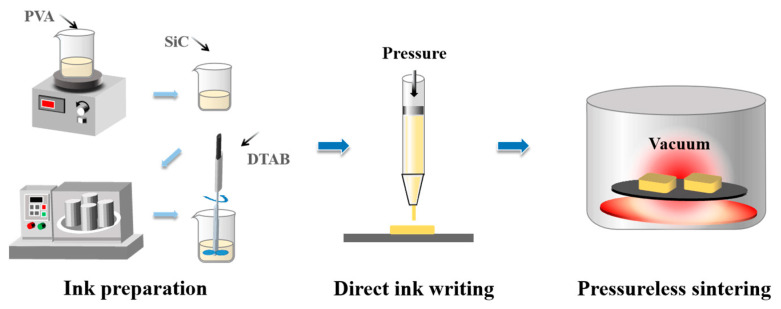
Fabrication process of porous SiC ceramics: ink preparation, direct-ink-writing-based 3D printing, and pressureless sintering.

The fabrication process of porous SiC ceramics contains three steps: ink preparation, direct-ink-writing (DIW)-based 3D printing, and pressureless sintering, as illustrated in Figure 1.

#### 2.2.1. Ink Preparation

The SiC powder loading of the ink we prepared was 35 vol%. Firstly, the pre-made suspension was prepared by mixing PVA and PEG into the deionized water, followed by magnetic stirring. Next, the premix powder prepared by mixing SiC powder, MC, Al_2_O_3_ powder, Y_2_O_3_ powder, and SiO_2_ powder was added into the suspension as sintering aids in a mass ratio of 7:2:1. After that, the mixture was continuously stirred in a planetary mill (QM-3SP2, Naijing University Instrument Plant, Naijing, China) at 400 r/min for 3 h to obtain the ink. Finally, the foam agent DTAB was added to the ink, and the foam ink was achieved by vigorously mixing at a speed of 1000 r/min for 5 min with a small mixer (LC-OES-120SH, Shanghai Lichen Bonsey Instrument Technology Co., Ltd., Shanghai, China). The compositions used for the printable SiC inks are listed in Table 1.

#### 2.2.2. Direct-Ink-Writing-Based 3D Printing

SiC green bodies were formed by a DIW-based 3D-printing system (Suzhou Fanbo Additive Manufacturing Technology Co., Ltd., Suzhou, China) equipped with a piston extrusion unit with nozzle sizes of 0.8 mm, 1.0 mm, and 1.5 mm. Other main 3D-printing parameters were: the printing speed was 10 mm/s, and the layer height was 100% of the nozzle diameters. The printed samples were brushed with glycerin on the surface to avoid cracking during drying and placed in an environment of 25 °C for 36 h.

#### 2.2.3. Pressureless Sintering

After complete drying, the DLP 3D-printed SiC green bodies were placed in a graphite furnace (ZT-70-23Y, Shanghai Chenxin Furnace Co., Ltd., Shanghai, China) and pressurelessly sintered in a vacuum environment. The heat program was set to be 10 °C/min before 1200 °C and then sintered to 1350–1650 °C with a heating rate of 2 °C/min and a dwelling time of 2 h in the furnace. After that, porous SiC-sintered ceramics were obtained.

### 2.3. Characterization

**Figure 2:**
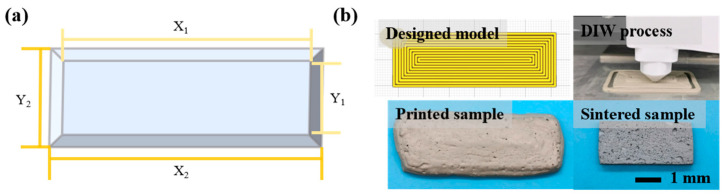
(**a**) Schematic diagram of sample shape; (**b**) designed model, direct-ink-writing 3D-printing process, and images of the samples after printing and sintering.

In this work, a cuboid CAD model of 20 mm (X_0_) × 60 mm (Y_0_) × 5 mm was designed using software. In order to quantify the dimensional accuracy of the samples, a digital caliper was used to measure their top and bottom dimensions, as shown in Figure 2a. The concepts of accuracy ratio (AR) and dimensional deviation ratio (DDR) were adopted with the following equations:

(1) $$PA=\frac{M_{1}-M_{0}}{M_{0}}\times 100\%$$

(2) $$DDR=\frac{M_{2}-M_{1}}{M_{1}}\times 100\%$$

where *M* was X or Y, *M*_1_ was the measured dimension of the top surface, and *M*_2_ was the measured dimension of the bottom surface. The open porosity of the sample was determined by using the Archimedes method. The sample size before and after sintering was measured with a digital caliper to calculate the shrinkage rate of sintering.

The morphology and microstructure of the samples were observed by using a scanning electron microscope (SEM, JSM-7500F, Hitachi Co., Tokyo, Japan). The X-ray diffraction (XRD, Holland, Cukα = 1.5418 Å) was used to characterize the crystal phases of the green body and samples at different sintering temperatures. The distribution data of pore size were measured by a high-performance automatic mercury-injection instrument (Autopore IV 9500, Micromeritics instrument CORP., Norcross, GA, USA). Compression tests were performed using a universal testing machine (Instron Legend 2367 testing system, Norwood, MA, USA) with a cross-head displacement rate of 0.5 mm/min. The test samples were cut into 10 ± 2 mm squares, and the stress loading was in the Z-direction of the printed sample. At least three tests for each group were performed.

The complex permittivity of porous SiC ceramics sintered at 1350–1650 °C was measured by using the waveguide method in the 6–18 GHz band using a vector network analyzer (KAA1171135, Electronic Technology Group 41 companies, Beijing, China). The samples were carefully ground to sizes of 34.7 mm × 15.7 mm, 22.9 mm × 10.1 mm, and 15.8 mm × 7.9 mm, and the thickness was limited to 4 mm in the thin section. The reflection loss (*R_L_*), which represents the absorption capacity of EM waves, can be calculated using transmission theory, as shown in the following equation [43]:

(3) $$Z_{in}=Z_{0}\sqrt{\frac{\mu }{\varepsilon }}\tan h\left[j\frac{2\pi }{c}\sqrt{\mu \pi }fd\right]$$

(4) $$R_{L}=20{log}_{10}\left|\frac{Z_{in}-Z_{0}}{Z_{in}+Z_{0}}\right|$$

where *Z_in_* is the input impedance, *Z*_0_ is the impedance of air, *c* is the light velocity, *f* is the frequency of EM wave, and *μ*, *ε*, and *d* are the relative permeability, relative permittivity, and thickness, respectively.

## 3. Results and Discussion

### 3.1. SiC Foam Ink for DIW-Based 3D Printing

**Figure 3:**
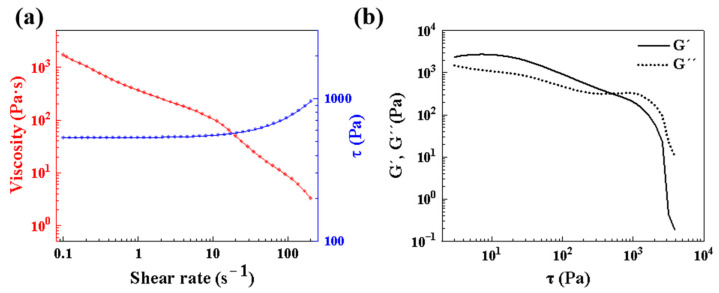
Rheological data of the SiC foam ink: (**a**) the steady shear viscosity, (**b**) the storage moduli (*G′*), and the loss moduli (*G″*).

The viscosity of the SiC foam ink decreased as the shear rate increased, exhibiting a shear-thinning behavior for extrusion of the ink by DIW-based 3D printing, as shown in Figure 3a. In addition, DIW requires the ink to be able to retain shape and self-supporting properties, which requires it to have an adequate initial storage modulus and yield stress to achieve. The results of the oscillatory rheological measurements are shown in Figure 3b. These results showed that the storage modulus (*G′*) dominated at lower shear stresses, while the loss modulus (*G″*) became more relevant at higher shear stresses after crossing the intersection point defined as the yield stress (*τ_y_*). The G′ and *τ_y_* values of the ink were 2.7 kPa and 490 Pa, respectively, which showed adequate viscoelasticity values [39]. Following the as-designed CAD model, the foam ink could be successfully printed by DIW-based 3D printing, as shown in Figure 2b.

### 3.2. SiC Green Bodies

**Figure 4:**
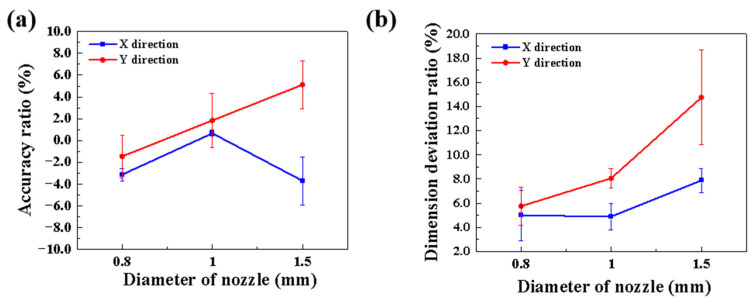
(**a**) accuracy ratio (AR) and (**b**) dimensional deviation ratio (DDR) of the SiC green bodies for different nozzle diameters.

Table 2 lists the dimensional parameters of the SiC green parts, and Figure 4 gives the accuracy ratio (AR) and dimensional deviation ratio (DDR) of the SiC green bodies for different nozzle diameters. The ratios of printing accuracy and dimensional deviation of the SiC green bodies in the *xy*-direction were lower when the nozzle diameter was small, and the difference in printing accuracy was related to the combined effect of the ink exhibiting an expansion characteristic similar to that of the pure polymer ink after the extrusion [44] and drying process of the material. However, when the nozzle diameter was too wide, the expansion characteristic of the ink was more obvious, and the ratios of printing accuracy and dimensional deviation exceeded 4%. Dimensional deviations arose because of the collapse of the foam ink, and it was found that the larger the diameter of the nozzle in the same place, the more obvious the collapse. Therefore, choosing a nozzle diameter of less than 1.5 mm facilitated the molding process. Meanwhile, the difference in data for different directions indicated that the expansion characteristics of the ink in the printing process were also related to the sample size.

**Figure 5:**
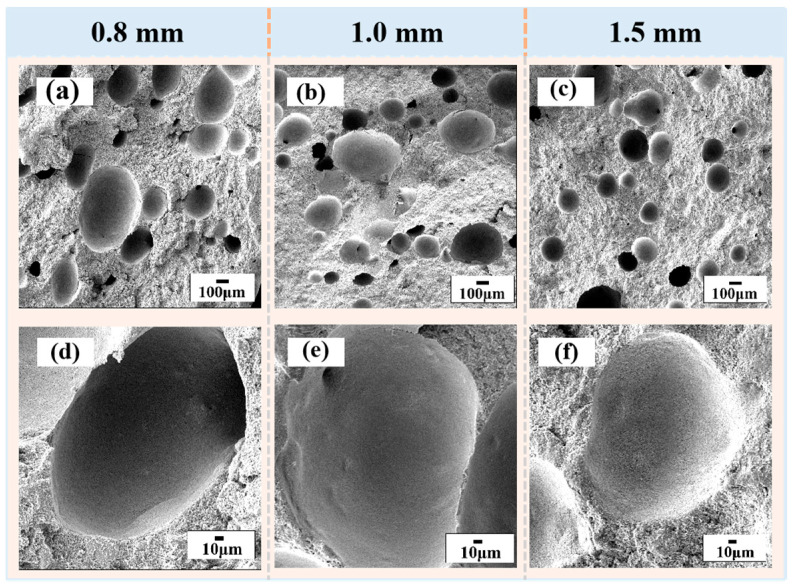
SEM micrographs of the SiC green bodies for different nozzle diameters: (**a**,**d**) the cross-sectional views of the 0.8 mm diameter, (**b**,**e**) the cross-sectional views of the 1.0 mm diameter, and (**c**,**f**) the cross-sectional views of the 1.5 mm diameter.

Figure 5 further exhibits the microstructure of the SiC green bodies for different nozzle diameters. The cross-sectional microstructures of the samples are shown in Figure 5a–d, where the SiC particles were combined by the binder. The size of the pores formed by the direct foaming method was approximately 100–200 μm, and most of them were spherical closed pores. The sample prepared by extrusion with the 1.5 mm nozzle diameter had the largest number of pores, and most of them were smaller pores with a diameter of approximately 100 μm, while the number of pores in the sample prepared with the 0.8 mm nozzle diameter decreased, and some of them turned into oval or larger pores with a diameter of approximately 200 μm. This was due to the increased stress on the filaments during printing as the nozzle diameter decreased and the phenomena of bubble breakage, deformation, and merger increased. In summary, combining formability and microstructure, we selected a nozzle diameter of 1.0 mm to carry out the follow-up work.

### 3.3. Porous SiC-Sintered Ceramics

#### 3.3.1. Phase Analysis and Microstructure Observation

**Figure 6:**
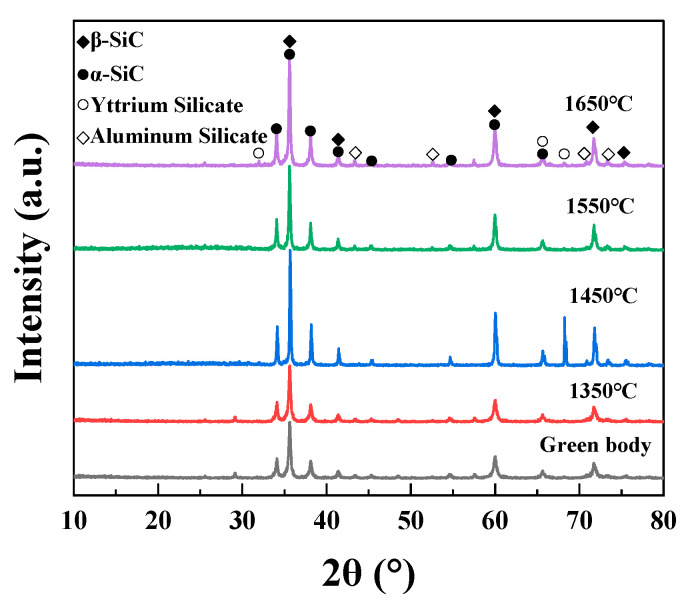
XRD patterns of the SiC green body and the porous SiC-sintered ceramics sintered at different sintering temperatures.

**Figure 7:**
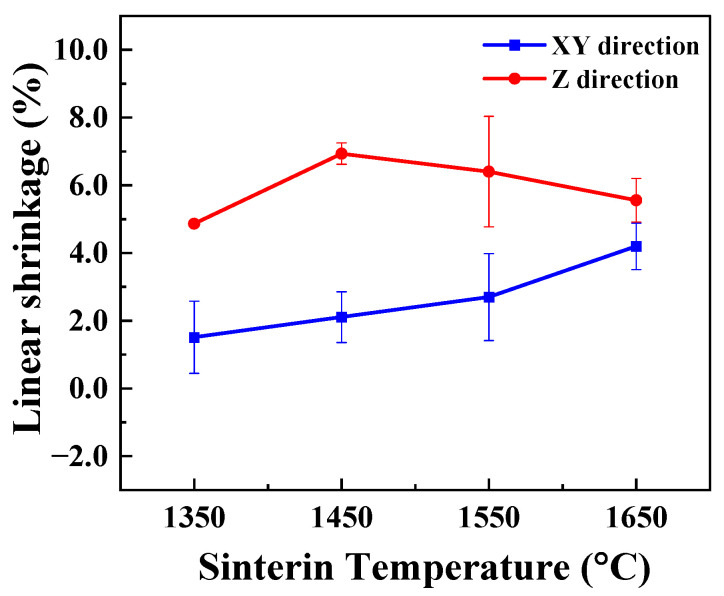
Shrinkage of the SiC green body and the porous SiC-sintered ceramics sintered at different sintering temperatures.

Figure 6 shows the XRD patterns of the SiC green body and the porous SiC-sintered ceramics sintered at different sintering temperatures. As shown in Figure 6, the as-sintered porous SiC ceramics consisted mainly of α-SiC (PDF card No. 49-1428) and β-SiC (PDF card No. 29-1129), and the results indicated that SiC ceramics were successfully fabricated. Additionally, the yttrium silicate and aluminum silicate phases were also observed in the samples when the sintering temperature exceeded 1450 °C, which implied a reactive sintering between the added sintering aids. These results proved that the composite additives could effectively reduce the sintering temperature of porous SiC ceramic [45]. We also investigated the variation in shrinkage in porous SiC ceramics sintered at different sintering temperatures. The shrinkage percentages were 1.51%, 4.86%, 4.20%, and 5.56% when the sintering temperatures were 1350 °C, 1450 °C, 1550 °C, and 1650 °C, respectively, as shown in Figure 7. The volume changes of the samples before and after sintering were relatively small, which was attributed to the fact that the sintering mechanism between the particles of the solid-state-sintered porous SiC ceramic was mainly surface diffusion [46]. It was also observed that the line shrinkage in the *xy*-direction was slightly lower than that in the *z*-direction for all ceramics. Since the layer-by-layer printing principle governs the DIW-based 3D-printing process, the difference in the different directions might be due to the inconsistency between the stacking density within the layers and between the layers [47].

**Figure 8:**
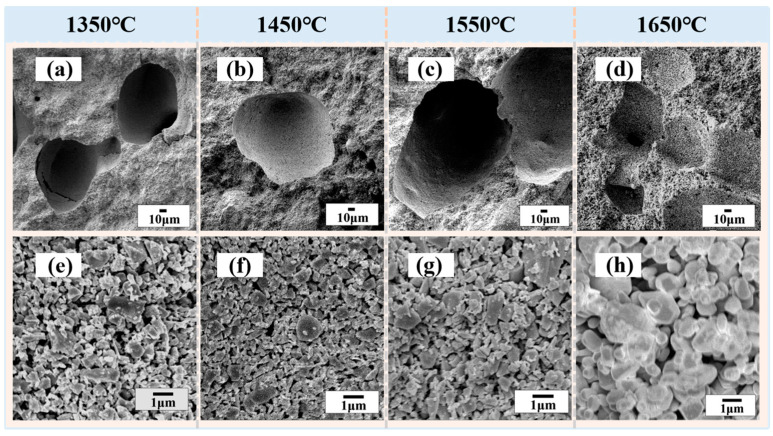
Cross-section SEM micrographs of the porous SiC ceramics sintered at different sintering temperatures: (**a**,**e**) 1350 °C, (**b**,**f**) 1450 °C, (**c**,**g**) 1550 °C, and (**d**,**h**) 1650 °C.

**Figure 9:**
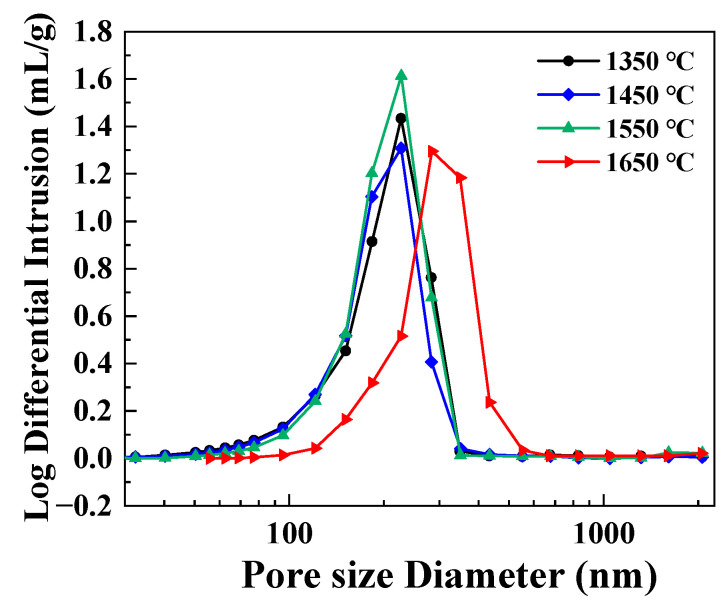
Pore-size distributions of the porous SiC ceramics sintered at different sintering temperatures.

The variations in the morphology of the porous SiC-sintered ceramics are shown in Figure 8. The cross-sectional microstructures of the samples are shown in Figure 8a–d. It was observed that when the temperature exceeded 1550 °C, the edges of the closed pores in the material were broken. This took place because the surface diffusion between particles was favored by temperature. As shown in Figure 8e–h, many grains smaller than 1 μm could be observed on the fracture surface when sintered at different temperatures. As the temperature increased, the surface of the grains appeared different. When the sintering temperature was no more than 1550 °C, the grain morphology was irregular and similar to that of the un-sintered grains; thus, the closed pore structure and the inter-grain pores remained stable at this temperature. After sintering at 1650 °C, the shape of the grains was no longer sharp, while the grains closely contacted each other and the phenomenon of neck growth occurred. The grain size increased, the volume shrinkage intensified, and the closed pore structure ruptured. To obtain further verification, we investigated the variation in pore-size distribution with different sintering temperatures, as shown in Figure 9. These results showed that the average pore size of the samples did not exceed 230 nm when the sintering temperature was less than 1550 °C but increased to 284 nm as the sintering temperature increased to 1650 °C. Combined with Figure 8, it was indicated that, as the sintering temperature increased, the sintered neck started to grow, the interparticle binding surface started to migrate, partial aggregation occurred, and, as a result, the interparticle pore size became slightly larger. The differences between them indicated that the pore morphology of porous SiC samples could be controlled by changing the sintering temperature.

#### 3.3.2. Mechanical Properties

**Figure 10:**
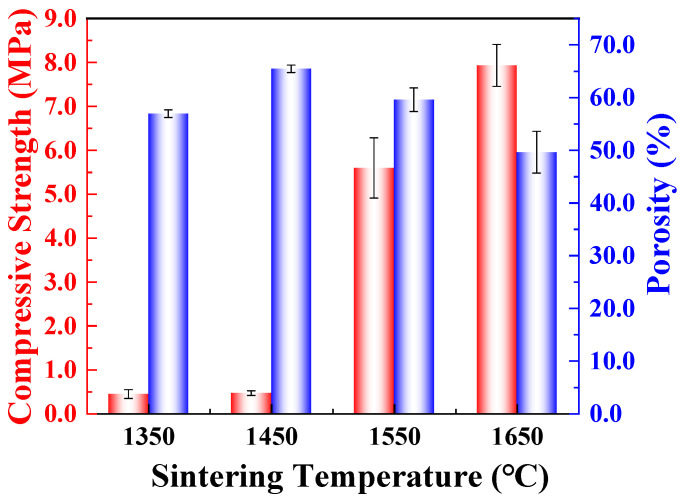
Open porosity and compressive strength of the porous SiC ceramics sintered at different sintering temperatures.

**Figure 11:**
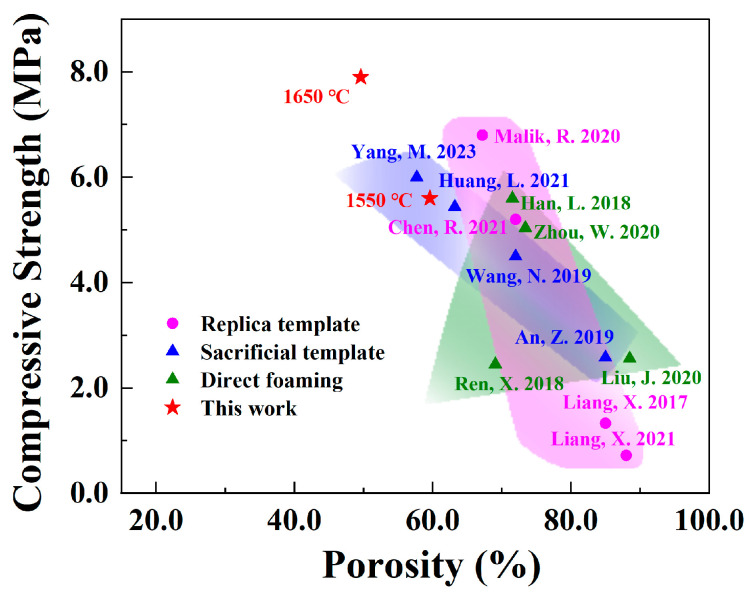
Comparison of the mechanical properties of porous SiC ceramics prepared by different methods [23,48,49,50,51,52,53,54,55,56,57,58].

The open porosity and compressive strength of the porous SiC ceramics sintered at different sintering temperatures are presented in Figure 10. The porosity of the porous SiC-sintered ceramics tended to increase and then decrease as the sintering temperature increased. The porous SiC ceramic sintered at 1450 °C had the highest porosity (65.4%), whereas the porosity of the porous SiC ceramic sintered at 1650 °C significantly decreased to 49.6%. This was due to the increased liquid phase during the sintering process, which led to the densification of the SiC particles and the increase in linear shrinkage shown in Figure 3. Similarly, the degree of particle densification was very important in influencing the mechanical properties of the ceramics. In Figure 10, the compressive strength of the porous SiC ceramics sintered at 1550 °C and 1650 °C exhibited a very sharp increase (from 0.5 MPa to a maximum of 7.9 MPa) compared with the porous SiC ceramics sintered at 1350 °C and 1450 °C. The rapid increase in compressive strength also indicated that, when the sintering temperature was higher than 1450 °C, the liquid phase formed by the sintering aid facilitated the sintering process, whereby the SiC ceramic particles changed from their original loosely packed state and became tightly bound to each other. The mechanical properties of the porous SiC ceramics were also compared with those of other reported porous ceramics, as shown in Figure 11 [23,48,49,50,51,52,53,54,55,56,57,58]. It was demonstrated that the porosity and compressive strength of the porous SiC ceramics prepared by different methods differed widely, and the compressive strength decreased with the increase in porosity. Although the porous SiC ceramics prepared in this study had a slightly lower porosity than traditional direct-foaming methods, their compressive strengths were greater than 5 MPa; hence, they were at the superior level. The better the mechanical properties of absorbing materials as functional materials under the aerospace covering, the more widely applied potential there is. The samples sintered at 1650 °C exhibited excellent compressive strength in porous ceramics. Certainly, the improvement in porosity and the maintenance of mechanical properties still need to be investigated in depth.

#### 3.3.3. Electromagnetic (EM) Wave Absorption Performance

**Figure 12:**
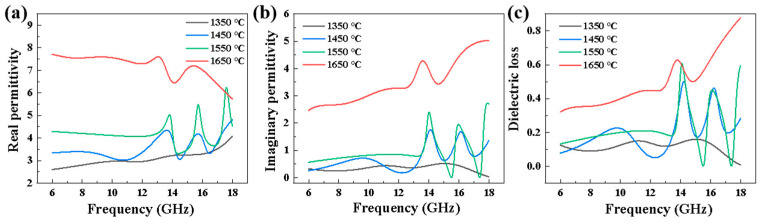
Dielectric properties of the porous SiC ceramics sintered at different sintering temperatures: (**a**) real permittivity, (**b**) imaginary permittivity, and (**c**) dielectric loss.

**Figure 13:**
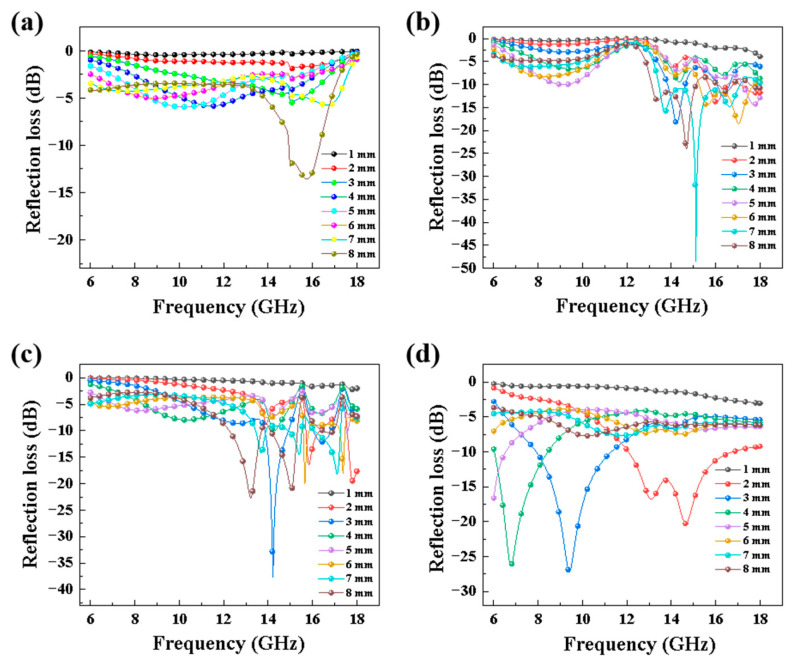
Reflection loss with different thicknesses of the porous SiC ceramics sintered at (**a**) 1350 °C, (**b**) 1450 °C, (**c**) 1550 °C, and (**d**) 1650 °C.

Due to the fact that SiC ceramics are diamagnetic, we only focused on their complex permittivity. Figure 12 shows the dielectric properties of porous SiC ceramics sintered at different sintering temperatures in the frequency range of 6–18 GHz. The real permittivity (*ε*’) represented the storage capability of the electric energy, and the imaginary permittivity (*ε*”) represented the loss capability. The tangent loss (tan *δ = ε*”/*ε*’) could be used to estimate the receding capabilities [59]. With the increase in sintering temperature, the values of *ε*’, *ε*”, and tan *δ* similarly increased, and the average value increased from 3.07 to 7.20, from 0.35 to 3.48, and from 0.11 to 0.49, respectively, which meant that the sintering temperature was favorable to improve the dielectric permittivity and loss tangent of the SiC material. These results could be attributed to the tightly bound heterogeneous interface between the SiC particles and the sintering aid, which enhanced the interfacial polarization [60]. Porous SiC ceramics contained air, which could improve the impedance match between the air and the absorbing material, resulting in increased loss capability. The reflection loss (*R_L_*) was the most intuitive parameter to accurately evaluate the absorption performance, and Figure 13 showed the *R_L_* values of samples with different thicknesses prepared at different sintering temperatures. Typically, the *R_L_* values of an excellent absorbing material should be less than −10 dB, which means that its EM wave absorption efficiency is more than 90%. In addition, when the *R_L_* value is less than −10 dB, the corresponding frequency range is defined as the effective absorption bandwidth (EAB), which is also an important factor to evaluate the absorption performance. It was observed that the *R_L_* values of porous SiC ceramics sintered at 1350 °C were hardly less than −10 dB, and the minimum *R_L_* value corresponding to 8.0 mm thickness reached −13.6 dB at 15.7 GHz, with a corresponding EAB value of 1.4 GHz. For the porous SiC ceramics sintered at 1450 °C, the minimum *R_L_* value corresponding to 7.0 mm thickness reached −48.9 dB at 15.1 GHz, with a corresponding EAB value of 3.7 GHz. When the sintering temperature was 1550 °C, the minimum *R_L_* value corresponded to a thinner thickness of 3.0 mm, with a value corresponding to −37.7 dB at 14 GH, and the EAB value was 1.8 GHz. The minimum *R_L_* value of porous SiC ceramics sintered at 1650 °C was −27.1 dB with a thickness of 3 mm at 9.4 GHz, with a corresponding EAB value of 3.4 GHz. It can be found that the permittivity and reflectivity of porous materials decreased with increasing porosity, which was due to the closed pore structure providing multiple reflections and increasing the loss path and to the absorbed EM energy being more readily converted to thermal energy, resulting in enhanced absorption performance [18,61]. Considering the practical applications, we needed to focus on evaluating the absorbing properties of materials with sintering temperatures of 1550 °C and 1650 °C. It is notable that the samples sintered at 1650 °C showed relatively favorable absorption properties at different frequency bands. While the sample sintered at 1550 °C had a better minimum *R_L_* value, the sample sintered at 1650 °C would only require a thickness of 2 mm to maintain a minimum R_L_ value lower than −25 dB, and the overall EAB was better than the former.

## 4. Conclusions

In this paper, we produced porous SiC ceramics by using the direct-foaming technique combined with direct-ink-writing (DIW)-based 3D printing and investigated the effects of sintering temperature on their mechanical properties and electromagnetic (EM) wave performance in detail. The main conclusions are summarized as follows:

(1) As the diameter of the nozzle increased, the formability of the porous SiC and the bubble breakage decreased. It was essential to select a suitable diameter that kept the microscopic pores closed while maintaining a certain level of printing accuracy.
(2) Sintering temperature had a large influence on the shrinkage, porosity, and compressive strength of porous SiC ceramics. As the sintering temperature increased, the porosity decreased while the compressive strength improved. The porous SiC ceramic sintered at 1650 °C exhibited the most optimal compressive strength (7.9 MPa).
(3) The dielectric permittivity and loss tangent of the porous SiC ceramics improved as the sintering temperature increased. Due to its highest porosity (65.4%), the porous SiC ceramic prepared at 1450 °C exhibited the most excellent electromagnetic absorption properties.
(4) Combined with the mechanical properties and wave absorption properties, the porous SiC ceramic sintered at 1650 °C was more capable of meeting the practical requirements of engineering applications, as evidenced by the minimum *R_L_* value of −27.1 dB at a smaller thickness and the EAB value of 3.4 GHz that was better than that of the samples sintered at 1550 °C.

In this study, the potential of direct foaming combined with direct-ink-writing-based 3D printing to prepare porous SiC ceramics for high strength and excellent EM wave absorption has been demonstrated.

## Figures and Tables

**Table 1:** Compositions of the printable SiC inks.

SiC Loading of the Ink, vol%	Relative to SiC Mass, wt%						MC Relative to the Distilled Water Mass, wt%
	PVA	PEG	Al_2_O_3_	Y_2_O_3_	SiO_2_	DTAB	
35	1.0	0.56	7.0	2.0	1.0	2.0	1.5

**Table 2:** The dimensional parameters of the SiC green parts.

Nozzle Diameter (mm)	*X*_1_ (mm)	*Y*_1_ (mm)	*X*_2_ (mm)	*Y*_2_ (mm)
0.8 mm	58.12 ± 0.34	19.71 ± 0.40	61.02 ± 0.99	20.84 ± 0.46
1.0 mm	60.39 ± 0.17	20.37 ± 0.50	63.35 ± 0.85	22.01 ± 0.61
1.5 mm	57.77 ± 1.32	21.02 ± 0.44	62.35 ± 0.87	24.11 ± 0.32

## Data Availability

Available on request.

## References

[1] Zeng X., Cheng X., Yu R., Stucky G. (2020). Electromagnetic microwave absorption theory and recent achievements in microwave absorbers. Carbon.

[2] Li Q., Zhang Z., Qi L., Liao Q., Kang Z., Zhang Y. (2019). Toward the application of high frequency electromagnetic wave absorption by carbon nanostructures. Adv. Sci..

[3] Li D., Jia D., Yang Z., Zhou Y. (2021). Principles, design, structure and properties of ceramics for microwave absorption or transmission at high-temperatures. Int. Mater. Rev..

[4] Liu C., Yu D., Kirk D.W., Xu Y. (2017). Electromagnetic wave absorption of silicon carbide based materials. RSC Adv..

[5] Mu Y., Zhou W., Hu Y., Ding D., Luo F., Qing Y. (2015). Enhanced microwave absorbing properties of 2.5D SiC_f_/SiC composites fabricated by a modified precursor infiltration and pyrolysis process. J. Alloys Compd..

[6] Yang L., Liu H., Zu M. (2018). Enhanced microwave-absorbing property of precursor infiltration and pyrolysis derived SiC_f_/SiC composites at X band: Role of carbon-rich interphase. J. Am. Ceram. Soc..

[7] Dou Y., Li J., Fang X., Jin H., Cao M. (2014). The enhanced polarization relaxation and excellent high-temperature dielectric properties of N-doped SiC. Appl. Phys. Lett..

[8] Nandihalli N., Gregory D., Mori T. (2022). Energy-Saving Pathways for Thermoelectric Nanomaterial Synthesis: Hydrothermal/Solvothermal, Microwave-Assisted, Solution-Based, and Powder Processing. Adv. Sci..

[9] Li Y., Liu X., Nie X., Yang W., Wang Y., Yu R., Shui J. (2019). Multifunctional Organic-Inorganic Hybrid Aerogel for Self-Cleaning, Heat-Insulating, and Highly Efficient Microwave Absorbing Material. Adv. Funct. Mater..

[10] Chen J., Liu M., Yang T., Zhai F., Hou X., Chou K. (2017). Improved microwave absorption performance of modified SiC in the 2–18 GHz frequency range. CrystEngComm.

[11] Lv X., Ye F., Cheng L., Zhang L. (2022). 3D printing “wire-on-sphere” hierarchical SiC nanowires/SiC whiskers foam for efficient high-temperature electromagnetic wave absorption. J. Mater. Sci. Technol..

[12] Ye F., Zhang L., Yin X., Liu Y., Cheng L. (2013). Dielectric and electromagnetic wave absorbing properties of two types of SiC fibres with different compositions. J. Mater. Sci. Technol..

[13] Meng S., Guo X., Jin G., Wang Y., Xie S. (2011). Preparation and microwave absorbing properties of SiC microtubes. J. Mater. Sci..

[14] Xiao T., Kuang J., Pu H., Zheng Q., Lu Y., Liu W., Cao W. (2021). Hollow SiC microtube with multiple attenuation mechanisms for broadband electromagnetic wave absorption. J. Alloys Compd..

[15] Dong S., Zhang X., Zhang D., Sun B., Yan L., Luo X. (2018). Strong effect of atmosphere on the microstructure and microwave absorption properties of porous SiC ceramics. J. Eur. Ceram. Soc..

[16] Xiang Z., He Q., Wang Y., Yin X. (2022). Hollow porous SiC spheres prepared by in-situ reaction with efficient microwave absorption. Ceram. Int..

[17] Liang C., Wang Z. (2019). Eggplant-derived SiC aerogels with high-performance electromagnetic wave absorption and thermal insulation properties. Chem. Eng. J..

[18] Liu C., Yu D., Kirk D., Xu Y. (2016). Porous silicon carbide derived from apple fruit with high electromagnetic absorption performance. J. Mater. Chem. C.

[19] Wu R., Zhou K., Yue C., Wei J., Pan Y. (2015). Recent progress in synthesis, properties and potential applications of SiC nanomaterials. Prog. Mate. Sci..

[20] Tuci G., Liu Y., Rossin A., Guo X., Pham C., Giambastiani G., Pham-Huu C. (2021). Porous silicon carbide (SiC): A Chance for improving catalysts or just another active-phase carrier?. Chem. Rev..

[21] Chen Y., Wang N., Ola O., Xia Y., Zhu Y. (2021). Porous ceramics: Light in weight but heavy in energy and environment technologies. Mat. Sci. Eng. R.

[22] Wei H., Cheng L., Shchukin D. (2020). Effect of Porous Structure on the Microwave Absorption Capacity of Soft Magnetic Connecting Network Ni/Al_2_O_3_/Ni Film. Materials.

[23] Huang L., Qin H., Hu T., Xie J., Guo W., Gao P., Xiao H. (2021). Fabrication of high permeability SiC ceramic membrane with gradient pore structure by one-step freeze-casting process. Ceram. Int..

[24] Jackowski M., Małek M. (2023). A multi-site study of a new cement composite brick with partial cement substitutes and waste materials. Case Stud. Constr. Mat..

[25] Liang C., Wang Z., Wu L., Zhang X., Wang H., Wang Z. (2017). Light and strong hierarchical porous SiC foam for efficient electromagnetic interference shielding and thermal insulation at elevated temperatures. ACS Appl. Mater. Interfaces.

[26] Cui Y., Cai J., Li Z., Jiao Z., Hu L., Hu J. (2022). Effect of Porosity on Dynamic Response of Additive Manufacturing Ti-6Al-4V Alloys. Micromachines.

[27] Rahmatabadi D., Soltanmohammadi K., Aberoumand M., Soleyman E., Ghasemi I., Baniassadi M., Abrinia K., Bodaghi M., Baghani M. (2022). Development of Pure Poly Vinyl Chloride (PVC) with Excellent 3D Printability and Macro-and Micro-Structural Properties. Macromol. Mater. Eng..

[28] Rahmatabadi D., Ghasemi I., Baniassadi M., Abrinia K., Baghani M. (2022). 3D printing of PLA-TPU with different component ratios: Fracture toughness, mechanical properties, and morphology. J. Mater. Res. Technol..

[29] Li L., Liu W., Wang Y., Zhao Z. (2023). Mechanical performance and damage monitoring of CFRP thermoplastic laminates with an open hole repaired by 3D printed patches. Compos. Struct..

[30] Moradi M., Aminzadeh A., Rahmatabadi D., Rasouli S. (2021). Statistical and Experimental Analysis of Process Parameters of 3D Nylon Printed Parts by Fused Deposition Modeling: Response Surface Modeling and Optimization. J. Mater. Eng. Perform..

[31] Chen Z., Li Z., Li J., Liu C., Lao C., Fu Y., Liu C., Li Y., Wang P., He Y. (2019). 3D printing of ceramics: A review. J. Eur. Ceram. Soc..

[32] He R., Zhou N., Zhang K., Zhang X., Zhang L., Wang W., Fang D. (2021). Progress and challenges towards additive manufacturing of SiC ceramic. J. Adv. Ceram..

[33] Shahzad A., Lazoglu I. (2021). Direct ink writing (DIW) of structural and functional ceramics: Recent achievements and future challenges. Compos. Part B-Eng..

[34] Franchin G., Scanferla P., Zeffiro L., Elsayed H., Baliello A., Giacomello G., Pasetto M., Colombo P. (2017). Direct ink writing of geopolymeric inks. J. Eur. Ceram. Soc..

[35] Huang K., Elsayed H., Franchin G., Colombo P. (2020). 3D printing of polymer-derived SiOC with hierarchical and tunable porosity. Addit. Manuf..

[36] Guo Z., Yang R., Wang T., An L., Ren S., Zhou C. (2021). Cost-effective additive manufacturing of ambient pressure-dried silica aerogel. J. Manuf. Sci. Eng..

[37] Muth J., Dixon P., Woish L., Gibson L., Lewis J. (2017). Architected cellular ceramics with tailored stiffness via direct foam writing. Proc. Natl. Acad. Sci. USA.

[38] Román-Manso B., Muth J., Gibson L., Ruettinger W., Lewis J. (2021). Hierarchically porous ceramics via direct writing of binary colloidal gel foams. ACS Appl. Mater. Interfaces.

[39] Minas C., Carnelli D., Tervoort E., Studart A. (2016). 3D printing of emulsions and foams into hierarchical porous ceramics. Adv. Mater..

[40] Guo Z., An L., Lakshmanan S., Armstrong J., Ren S., Zhou C. (2022). Additive manufacturing of porous ceramics with foaming agent. J. Manuf. Sci. Eng..

[41] Ma S., Liu X., Fu S., Zhao S., He P., Duan X., Yang Z., Jia D., Colombo P., Zhou Y. (2022). Direct ink writing of porous SiC ceramics with geopolymer as binder. J. Eur. Ceram. Soc..

[42] Wang W., Bai X., Zhang L., Jing S., Shen C., He R. (2022). Additive manufacturing of Cs_f_/SiC composites with high fiber content by direct ink writing and liquid silicon infiltration. Ceram. Int..

[43] Yan J., Huang Y., Liu X., Zhao X., Li T., Zhao Y., Liu P. (2021). Polypyrrole-Based Composite Materials for Electromagnetic Wave Absorption. Polym. Rev..

[44] Ding G., He R., Zhang K., Xie C., Wang M., Yang Y., Fang D. (2019). Stereolithography-based additive manufacturing of gray-colored SiC ceramic green body. J. Am. Ceram. Soc..

[45] Zhao H., Liu Z., Yang Y., Liu X., Zhang K., Li Z. (2011). Preparation and properties of porous silicon carbide ceramics through coat-mix and composite additives process. Trans. Nonferrous Met. Soc. China.

[46] Liu J., Xiao H., Guo W., Gao P., Liang J. (2018). Spheroidization of SiC powders and their improvement on the properties of SiC porous ceramics. Ceram. Int..

[47] Liu S., Li M., Wu J., Chen A., Shi Y., Li C. (2020). Preparation of high-porosity Al_2_O_3_ ceramic foams via selective laser sintering of Al_2_O_3_ poly-hollow microspheres. Ceram. Int..

[48] Wang N., Liu Y., Zhang Y., Du Y., Zhang J. (2019). Control of pore structure during freeze casting of porous SiC ceramics by different freezing modes. Ceram. Int..

[49] Ren X., Ma B., Zhang Y., Zhu Q., Li D., Li S., Yuan L., Yu J., Liu G., Li H. (2018). Effects of sintering temperature and V_2_O_5_ additive on the properties of SiC-Al_2_O_3_ ceramic foams. J. Alloys Compd..

[50] Chen R., Jin X., Hei D., Lin P., Liu F., Zhan J., Lao D., Li M., Jia W., Shan Q. (2021). Enhanced mechanical strength of SiC reticulated porous ceramics via addition of in-situ chopped carbon fibers. J. Alloys Compd..

[51] Zhou W., Yan W., Li N., Li Y., Dai Y., Zhang Z. (2020). Fabrication and characterization of a mullite-foamed ceramic reinforced by in-situ SiC whiskers. Ceram. Int..

[52] Liang X., Li Y., Liu J., Sang S., Chen Y., Li B., Aneziris C. (2017). Improvement of the mechanical properties of SiC reticulated porous ceramics with optimized three-layered struts for porous media combustion. Ceram. Int..

[53] Han L., Wang J., Li F., Wang H., Deng X., Zhang H., Zhang S. (2018). Low-temperature preparation of Si_3_N_4_ whiskers bonded/reinforced SiC porous ceramics via foam-gelcasting combined with catalytic nitridation. J. Eur. Ceram. Soc..

[54] Malik R., Kim Y., Song I. (2020). High interfacial thermal resistance induced low thermal conductivity in porous SiC-SiO_2_ composites with hierarchical porosity. J. Eur. Ceram. Soc..

[55] Yang M., Zhang J., Zhang Y., Sun W., Shi Z. (2023). Permeability and corrosion resistance of porous SiOC-bonded SiC ceramics prepared by the preceramicpolymer. Int. J. Appl. Ceram. Technol..

[56] An Z., Zhang R., Fang D. (2019). Synthesis of monolithic SiC aerogels with high mechanical strength and low thermal conductivity. Ceram. Int..

[57] Liu J., Ren B., Rong Y., Lu Y., Zhao Y., Wang L., Xi X., Yang J., Huang Y. (2020). Ultralight and mechanically robust SiC foams with interconnected cellular architecture. Ceram. Int..

[58] Liang X., Li Y., Yan W., Wang Q., Tan F., He Z., Sang S. (2021). Preparation of SiC reticulated porous ceramics with high strength and increased efficient filtration via fly ash addition. J. Eur. Ceram. Soc..

[59] Dong Y., Fan X., Wei H., Hou Z., Li M., Qu Q., Yin X., Cheng L., Zhang L. (2020). A lightweight CNWs-SiO_2_/3Al_2_O_3_·2SiO_2_ porous ceramic with excellent microwave absorption and thermal insulation properties. Ceram. Int..

[60] Dai D., Lan X., Wu L., Wang Z. (2022). Designed fabrication of lightweight SiC/Si_3_N_4_ aerogels for enhanced electromagnetic wave absorption and thermal insulation. J. Alloys Compd..

[61] Wang S., Xiao N., Zhou Y., Ling Z., Li M., Qiu J. (2016). Lightweight carbon foam from coal liquefaction residue with broad-band microwave absorbing capability. Carbon.

